# Change in colorectal cancer (CRC) testing rates associated with the introduction of the first organized screening program in canton Uri, Switzerland: Evidence from insurance claims data analyses from 2010 to 2018

**DOI:** 10.1016/j.pmedr.2022.101851

**Published:** 2022-06-10

**Authors:** Sarah Bissig, Lamprini Syrogiannouli, Rémi Schneider, Kali Tal, Kevin Selby, Cinzia Del Giovane, Jean-Luc Bulliard, Oliver Senn, Cyril Ducros, Christian P.R. Schmid, Urs Marbet, Reto Auer

**Affiliations:** a Institute of Primary Health Care (BIHAM), University of Bern, Bern, Switzerland; b Center for Primary Care and Public Health (Unisanté), University of Lausanne, Lausanne, Switzerland; c Institute of Primary Care, University and University Hospital of Zurich, Zurich, Switzerland; d CSS Institute for Empirical Health Economics, Tribschenstrasse 21, Lucerne, Switzerland; e Division of Gastroenterology and Hepatology Cantonal Hospital of Uri, Altdorf, Switzerland

**Keywords:** colorectal cancer, screening, organized screening program, colonoscopy, fecal occult blood test, health insurance, claims data, testing rates, OSP, organized screening program, NB, neighboring cantons, CRC, colorectal cancer, FOBT, gFOBT, iFOBT, fecal occult blood test, guaiac or immunochemical based (also called FIT), Uri, the canton of Uri, SHS, swiss health survey, AL, Swiss analysis list for laboratory measures, TARMED, Swiss ambulant procedures codes, FSO, federal statistics office, PCG, pharmacy based cost groups

## Abstract

The first canton in Switzerland to implement an organized colorectal cancer screening program (OSP) was Uri. Starting in 2013, it offered 50–69-year-olds free testing with colonoscopy every 10 years or fecal occult blood test (FOBT) every 2 years. We tested the association between the OSP and testing rates over time.

We analyzed claims data of 50–69-year-olds from Uri and neighboring cantons (NB) provided by a large health insurance and complemented it with data from the OSP. We fitted multivariate adjusted logistic regression models to compare overall testing rates and by method (colonoscopy or FOBT/both) We computed the 2018 rate of the population up-to-date with testing (colonoscopy within 9 years/FOBT within 2 years).

Yearly overall testing rates in Uri increased from 8.7% in 2010 to 10.8% in 2018 and from 6.5% to 7.9% in NB. In Uri, the proportion tested with FOBT/both increased from 4.7% to 6.0% but decreased from 2.8% to 1.1% in NB. Testing by FOBT/both increased more between 2015 and 2018 than 2010–2012 in Uri than in NB (OR:2.1[95%CI:1.8–2.4]), it increased less for colonoscopy (OR:0.60[95%CI:0.51–0.70]), with no change in overall CRC testing (OR:0.91[95%CI:0.81–1.02]). In 2018 in Uri, 42.5% were up-to-date with testing (FOBT/both:9.2%, colonoscopy:35.7%); in NBs, 40.7% (FOBT/both:2.7%, colonoscopy:39%).

Yearly FOBT rates in Uri were always higher than in NB. Though the OSP in Uri was not associated with a greater increase in overall testing rates, the OSP was associated with increased FOBT.

## Highlights

• First colorectal cancer (CRC) screening program in Switzerland launched in one canton in 2013.
• Launched in the context of high prevalent opportunistic CRC testing.
• Led to an increase in fecal occult blood testing and not colonoscopy.
• Claims data analyses enable estimating the net effect of programs in general population.

## 1 Introduction

Colorectal cancer (CRC) screening can cut CRC mortality in half (Meester et al., 2015) and Switzerland, like the EU and the USA, recommends either colonoscopy every ten years or fecal occult blood test (FOBT) every two years for 50-to-69-year-olds in the average-risk population (Bibbins-Domingo et al., 2016, von Karsa, 2013; Bundesamt fuer Gesundheit: Nationale Strategie gegen Krebs 2014–2017, 2015). But in Switzerland, CRC screening is mostly opportunistic, though we know that organized CRC screening programs (OSP) effectively increase screening uptake and quality of care and ensure equal access to preventative healthcare (von Karsa, 2013). In 2000, CRC screening choices in Cantons Uri and Glarus were evaluated in a cohort study that invited 22,818 eligible residents for a free immunological FOBT (iFOBT), sigmoidoscopy/colonoscopy or a combination of both. Of those invited, 2731 elected to be tested (12% of the eligible population; 297 with FOBT; 278 with FOBT annually + sigmoidoscopy every 5 years and 2056 with colonoscopy/sigmoidoscopy only) (Marbet et al., 2008). Then in 2013, Uri launched Switzerland’s first OSP. That same year, Swiss insurances began reimbursing adults aged 50–69 for screening with either colonoscopy every 10 years or FOBT every 2 years and waived the deductible. (All Swiss residents have at least basic mandatory health insurance). The Uri OSP also waives the co-payment fee.

Uri’s OSP sends all 50-year-old inhabitants a letter explaining the program and inviting them to be screened for CRC. Eligible residents are encouraged to talk to their family physician about CRC screening and are offered an informed choice between an iFOBT, also called FIT, or a colonoscopy. The program organizers track participation rates to determine the effectiveness and reach of their invitations. But they cannot measure the overall effect of the program on cantonal screening rates because they do not know if participants who are screened within the program would have otherwise elected to be screened outside the program. Since opportunistic CRC screening and diagnostic testing is high in Switzerland compared to other countries (Braun, 2020) and continues to run in parallel to Uri’s OSP, determining the effect of the program on screening rates requires more representative data.

One type of data that can help determine overall testing rates in the whole population and specific subgroups is insurance claims data (Schenck et al., 2007, Fiscella et al., 2006, Gupta, 2013, Stock et al., 2011). This data, collected passively and continuously, captures changes in colonoscopy and FOBT testing rates before and after an OSP is implemented and for CRC testing performed within the OSP and opportunistic testing performed outside of the OSP (Wharam et al., 2016, Wharam et al., 2011, Mehta, 2015). Claims data is also timelier than survey data and collecting is less complex and expensive.

We aimed to assess the effect of the OSP in the canton of Uri on CRC testing rates over time and compared to neighboring cantons (NB) without an OSP, in a country with high prevalent opportunistic CRC testing. We used claims data from a large health insurance to describe yearly changes in CRC testing before and after Uri implemented its OSP, and to compare testing rates with NBs that lack a CRC OSP. We also contrasted the proportion of the population in Uri and NB that was up-to-date with CRC testing (colonoscopy within 9 years or FOBT within 2 years) in 2018.

## 2 Methods

### 2.1 Study setting

This is a set of yearly cross-sectional analyses and a cross-sectional analysis in 2018 of a prospective cohort of insurees of one large health insurance, all of whom reside in Uri or one of five NBs.

### 2.2 Data

We used data from a large Swiss health insurance (CSS) that contains about 1.6 million insurees per year (16% of the Swiss population; 20% of the Uri population) and represents the general population of all 26 cantons (Bischof and Schmid, 2018, Trottmann et al., 2012). The research center of CSS insurance (CSS Institute for Empirical Health Economics) extracted the raw data, which we then independently analyzed. We obtained data on outpatient health care over a 9-year study period (January 1, 2010, to December 31, 2018), including data on sociodemographic factors, insurance plan, health care use, associated costs from all health care settings, health status proxies, and ambulatory billing for CRC testing. Since recorded insurance claims cover almost all health care and pharmacy invoices, this data should be near complete, though it does not capture an estimated 1.7%−2.4% of out-of-pocket health care expenditures (Schmid, 2017).

Since in 2013 and 2014, FOBTs were free to participants in the Uri program and were not billed to health insurances, the cantonal health department provided the number of tests billed to Uri. We used this billing data to estimate testing rates in 2013–2014 and validate our findings overall.. The OSP only shared the total number of tests billed and Swiss law on human research prevented us from linking the individual FOBTs billed in the cantonal dataset to the CSS health insurance.

We also compared the testing rates we computed from claims data and OSP data to data from the Swiss Health Survey dataset (SHS), a population-based survey conducted every five years (Federal statistics office: Swiss Health Survey 2017, overview, 2018). (Appendix Table 2 for overview on used data sources; Appendix Table 6-8 for SHS data). Data was de-identified and anonymized so ethical approval was not required under the Swiss Human Research Act.

### 2.3 Inclusion and exclusion criteria

For the yearly cross-sectional analyses, we included those 50–69-years on the last day of the year who had been continuously enrolled in the same CSS basic insurance plan for 11 months or more that year. We excluded participants who died or moved during the study year (Appendix Table 4).

For our 2018 cross-sectional analysis of the proportion up-to-date with testing (colonoscopy within the last 9 years or FOBT within the last 2 years), we restricted the dataset to insurees continuously insured between 2010 and 2018. Since we were tracking participants aged 50–69 in 2010 over a 9-year period, we limited our analyses to participants aged 59–69 in 2018.

### 2.4 Geographic units

We compared insurees across Uri and NBs and used the same inclusion criteria for the populations of Lucerne, Nidwalden, Obwalden, Schwyz, and Glarus. We included these cantons because of a similar socio-demographic structure as well as areal and economic linkage. The residents of Glarus also participated in the 2000 cohort study (Marbet et al., 2008, Manser et al., 2012).

### 2.5 Outcome measures

We extracted billing codes associated with CRC testing methods. We used the Swiss analysis list for laboratory measures (AL) to identify billed FOBTs. The AL uses the same billing code for guiaiac-based FOBT (gFOBT) and iFOBT. We used the billing code for FOBT t without the possibility of distinguishing the type of test billed. We used Swiss ambulatory procedures codes (TARMED) to identify colonoscopies, sigmoidoscopies, and recto-sigmoidoscopies (Appendix Table 3). Since sigmoidoscopies are rarely performed in Switzerland, we classed them together with colonoscopies. We had no data on tests performed in inpatient settings. Billing data does not include reasons for testing (screening or diagnostic), but after 2014 the OSP used separate codes, so we could determine if tests were administered inside or outside the OSP.

In 2013 and 2014, Uri gave participants free iFOBT which were not billed to health insurances. But OSP conductors gave us data on overall program participation. We then used CSS data to compute the market share of CSS insurance among eligible people in Uri. Overall program participation and CSS market share allowed us to estimate FOBT testing rates inside the program and overall testing rates in Uri in 2013 and 2014 (Appendix Table 5).

### 2.6 Covariates

We collected demographic data (birth date, death date, gender, zip code) from CSS standard insuree data. Based on the list provided by the Federal Statistics Office (FSO), we converted zip codes to FSO community numbers to determine canton and residence (urban/intermediate/ rural). For each year, we extracted the participant’s deductible (in CHF), and health plan (free physician choice, family physician as gatekeeper, health maintenance organization [HMO] or telemedicine). Health insurance data in Switzerland does not include clinical diagnoses. Since pharmacy-based cost groups (PCGs) identify medications used to treat chronic diseases, we used these as a proxy for chronic disease, and extracted the number of PCGs for each enrollee (Lamers, 1999, Lamers and van Vliet, 2004, Lamers and Vliet, 2003).

### 2.7 Statistical analyses

To analyze yearly CRC testing rates, we defined: a) “any testing for CRC” as insurees with a bill for at least one test (FOBT or colonoscopy) in that calendar year; b) “FOBT/both” as insurees billed for one or more FOBTs with or without a colonoscopy; and c) “colonoscopy” as insurees who had had a colonoscopy and no FOBT that calendar year. People who had taken both tests were placed in the FOBT group because a positive FOBT likely led to a colonoscopy. (Marbet et al., 2008, Fischer et al., 2013).

We used descriptive statistics to characterize enrollees and calculated percentages of these characteristics for each study year, determining the proportions of people who had had a) any test for CRC, b) FOBT/both, and c) colonoscopy only. We did this for each year (2010 to 2018) and each geographical area (Uri vs NB). We then stratified results into two age groups (50–59 and 60–69) and differentiated between three different types of residence (urban/intermediate/rural). Patients with family physician, telemedicine or HMO insurance model plans were classed as managed care.

To compute the percentage of insurees in 2018 tested for CRC at recommended intervals in each geographical region, we restricted the dataset to those continuously insured from 2010 to 2018. We tracked insurees backward to identify bills for FOBT in 2018 or 2017 or any colonoscopy between 2010 and 2018. Insurees were a) up-to-date with any CRC testing if they had had at least one colonoscopy between 2010 and 2018 and/or any FOBT in 2017–2018.They were b) up-to-date with FOBT if they were billed for one or more FOBTs in 2017 or 2018, with or without a colonoscopy. And they were c) up-to-date with colonoscopy if they had had any colonoscopy between 2010 and 2018 and no FOBT in 2017–2018.

We fitted a multivariate adjusted logistic regression model to compute OR of any CRC testing (tested vs. not tested) and a multinomial regression model to compute OR of colonoscopy only, FOBT/both, and no test. Covariates of adjustment were age, gender, residence, comorbidities (PCGs), health insurance plan, and location (Uri vs NBs).

To test for an effect of the OSP on testing rates, we defined two time periods: 2010–2012 (before the program was implemented in 2013) and 2015–2018 (after the program was implemented). Data on FOBT were incomplete in the claims dataset in 2013 and 2014 because the canton did not bill the health insurance for FOBTs inside the program, so we excluded 2013–2014 from these analyses. We included year of testing as a categorical predictor and tested for an interaction between time period (2010–2012 vs 2015–2018) and canton. We further performed stratified analyses by gender to test gender differences in the effect of the OSP on testing rate.

The threshold for statistical significance for all analyses was p < 0.05. We used Stata Software for all statistical analyses.

## 3 Results

### 3.1 Yearly rates of CRC testing

For this analysis we included between 40,323 to 48,655 insurees aged 50–69 per year.

The proportion of older insurees and people living in rural regions was higher in Uri than in NBs and insurees in Uri were less likely to subscribe to a form of managed care, but this difference narrowed over the years (Table 1).

**Table 1:** Descriptive statistics of the included population (aged 50–69) in Uri and neighboring cantons^+^ every 2 calendar years (2010–2018); CSS database.

	2010		2012		2014		2016		2018	
	Uri	NB	Uri	NB	Uri	NB	Uri	NB	Uri	NB
Population (N)	1′891	38′432	1′875	39′166	1′996	41′590	2′089	43′614	2′176	46′479
Gender (=woman) % (CI)	50.3 (48.0–52.5)	50.0 (49.5–50-5)	50.3 (48.0–52.6)	50.0 (49.5–50.5)	50.4 (48.2–52.6)	49.9 (49.3–50.2)	49.2 (47.0–51.3)	49.8 (49.3–50.2)	49.6 (47.5–51.7)	49.8 (49.4–50.3)
Age % (CI)										
50–59	54.3 (52.0–56.5)	57.5 (57.0–57.9)	53.9 (51.7–56.2)	57.5 (57.1–58.0)	53.9 (51.7–56.1)	58.4 (58.9–58.9)	54.7 (52.6–56.8)	59.1 (58.6–59.5)	54.2 (52.1–56.3)	58.9 (58.4–59.3)
60–69	45.7 (43.5–48.0)	42.5 (42.0–43.0)	46.1 (43.8–48.3)	42.5 (42.0–42.9)	46.1 (43.9–48.3)	41.6 (41.1–42.0)	45.3 (43.2–47.4)	40.9 (40.5–41.4)	45.8 (43.7–47.9)	41.1 (40.7–41.6)
Residence^1^ % (CI)										
Urban	63.2 (61.0–65.4)	50.5 (50.0–51.0)	62.9 (60.7–65.0)	50.0 (49.6–50.5)	64.3 (62.2–66.4)	49.7 (49.2–50.1)	64.1 (62.0–66.1)	49.5 (49.0–50.0)	65.1 (63.0–67.1)	49.1 (48.6–49.6)
Intermediate	11.5 (10.2–13.0)	20.2 (19.8–20.6)	12.2 (10.8–13.7)	20.6 (20.2–21.0)	11.1 (9.8–12.5)	20.9 (20.5–21.3)	12.5 (11.1–13.9)	21.1 (20.7–21.5)	12.7 (11.4–14.2)	21.3 (20.9–21.6)
Rural	25.2 (23.3–27.2)	29.3 (28.9–29.8)	25.0 (23.1–27.0)	29.4 (28.9–29.8)	24.6 (22.8–26.5)	29.4 (29.0–30.0)	23.5 (21.7–25.4)	29.4 (29.0–29.9)	22.2 (20.5–24.0)	29.6 (29.2–30.0)
Managed care^2^ = Yes; % (CI)	28.7 (26.7–30.8)	49.0 (48.5–49.5)	35.2 (33.1–37.4)	56.6 (56.1–57.1)	46.4 (44.3–48.6)	61.5 (61.0–61.9)	55.2 (53.0–57.3)	65.8 (65.4–66.3)	63.9 (61.8–65.9)	70.1 (70.6–71.4)
PCG^3^ ≥ 1; % (CI)	35.5 (33.4–37.7)	36.1 (35.6–36.6)	37.5 (35.4–39.8)	37.5 (37.0–38.0)	36.2 (34.1–38.3)	38.1 (37.6–38.6)	35.8 (33.7–37.8)	37.9 (37.4–38.3)	35.2 (33.2–37.2)	36.9 (36.5–37.4)

Exclusion criteria: died, moved or changed insurance in that year + = Neighboring cantons; Glarus (GL), Lucerne (LU), Nidwalden (NW), Obwalden (OW), Schwyz (SZ) 1: determined from the postal code and a Federal Statistical Office of Switzerland (FSO) list 2:included this model: family physician, HMO, and telemedicine 3 pharmacy-based cost group; method of assessing chronic health conditions based on information about medication.

In Uri, overall testing rates increased from 8.7% in 2010 to 10.8% in 2018. In NBs it increased from 6.5% to 7.9. Testing with FOBT/both in Uri increased from 4.7% to 6.0% while it decreased from 2.8% to 1.1% in NBs. Colonoscopy rates in Uri increased from 4.1% to 4.8%, while it increased from 3.7% to 6.8% in NBs. In 2013, 2.8% of the included population participated in the program and took an FOBT. In 2018, this proportion rose to 4.2%. The rest who received FOBT were tested outside of the program (1.7% in 2018).

**Fig. 1:**
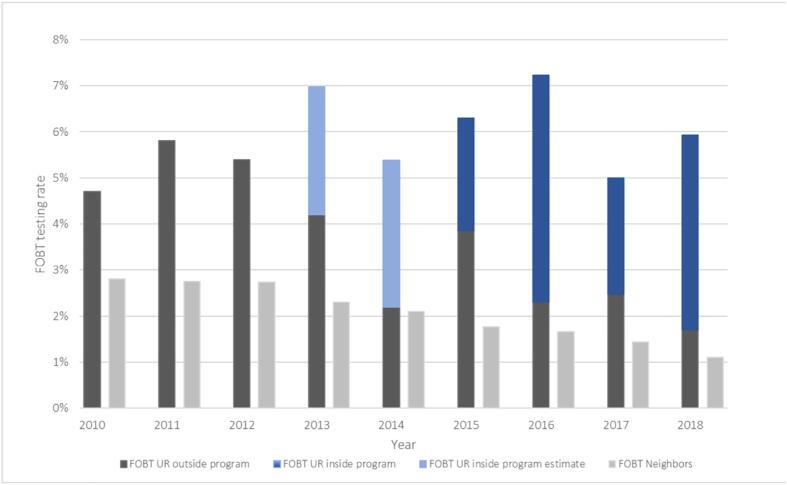
Yearly testing rates for FOBT only or FOBT and colonoscopy in Uri and its neighboring cantons^+^ among 50–69 year-olds 2010–2018; CSS Database Exclusion criteria: died, moved or changed insurance in that year. + neighboring cantons: Glarus (GL), Lucerne (LU), Nidwalden (NW), Obwalden (OW), Schwyz (SZ). FOBT UR inside: FOBT test within the program (assigned its own billing code after 2014). FOBT UR inside estimate: estimate of true testing rate based on data from the Uri program, when billing claims data was missing (these tests were free in Uri and not billed). FOBT UR outside: testing before or outside of the program, visible in bills after 2014. FOBT Neighbors: overall FOBT incidences in included neighboring canton where there were no organized screening programs.

**Fig. 2:**
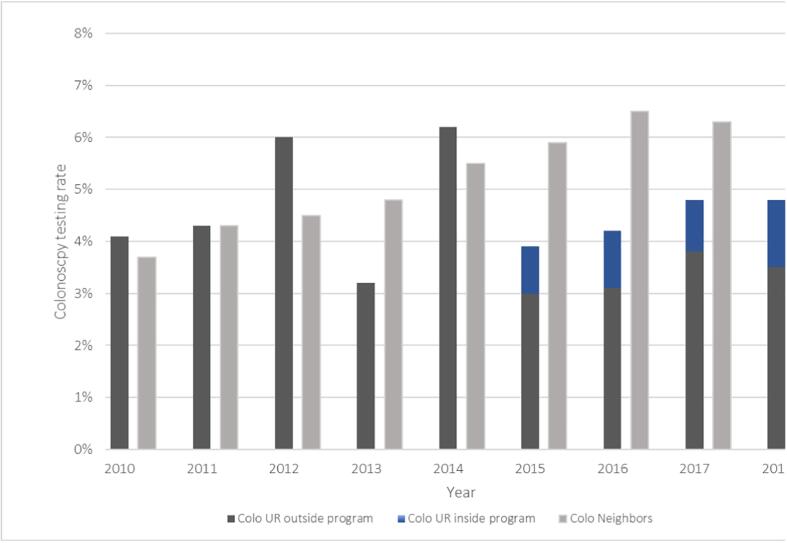
Yearly testing rates for colonoscopy only in Uri and neighboring cantons^+^ among 50–69 year olds from 2010 to 2018, CSS Database. Exclusion criteria: died, moved or changed insurance in that year. + neighboring cantons: Glarus (GL), Lucerne (LU), Nidwalden (NW), Obwalden (OW), Schwyz (SZ). Colo UR inside program: colonoscopy within the program, after 2014 assigned its own billing code. Colo UR outside program: testing before or outside of the program, visible in bills after 2014. Colo Neighbors: overall colonoscopies in included neighboring cantons, which had no organized screening programs.

2015 was the first year we could distinguish between colonoscopies performed within and outside the OSP. In 2015, 0.9% of eligible insurees had a colonoscopy within the program, increasing to 1.3% by 2018). The same year, 3.5% had a colonoscopy outside the program (Fig. 1, Fig. 2, Table 2).

**Table 2:** Probability of having had a) any test for colorectal cancer [fecal occult blood test (FOBT) or colonoscopy], b) FOBT or FOBT and colonoscopy or c) any colonoscopy and no FOBT among 50–69 year-olds in Uri and neighboring cantons (NB^)+^, by calendar year (2010–2018). Uri’s organized screening program for colorectal cancer started in 2013. CSS database years: 2010 to 2018.

	2010	2011	2012	2013	2014	2015	2016	2017	2018
Uri (N Population)	1891	1869	1875	1897	1996	2020	2089	2105	2176
Neighbors^+^ (N Population)	38′432	38′471	39′166	40′356	41′590	42′559	43′614	44′923	46′479
a) Overall testing rates									
Uri %(CI) N Test	8.7 (7.5–10.1) 165	10.1 (8.7–11.5) 188	11.4 (10.0–12.9) 214	10.1*(8.8–11.6) 192	11.6* (10.2–13.1) 231	10.2 (9.0–11.7) 207	11.4 (10.1–12.8) 238	9.7 (8.5–11.1) 205	10.8 (9.5–12.2) 235
NB % (CI) N Test	6.5 (6.3–6.8) 2′506	7.1 (6.8–7.3) 2′725	7.2 (7.0–7.5) 2′824	7.1 (6.8–7.3) 2′849	7.6 (7.3–7.8) 3′144	7.7 (7.4–7.9) 3′256	8.2 (8.0–8.5) 3′579	7.8 (7.5–8.0) 3′497	7.9 (7.7–8.2) 3′686
b) FOBT or both									
Uri Overall % (CI) N Test	4.7 (3.8–5.7) 88	5.8 (4.8–6.9) 108	5.4 (4.5–6.5) 101	7.0* (5.9–8.2) 132	5.4* (4.4–6.4) 107	6.3 (5.3–7.4) 127	7.3 (6.2–8.5) 152	5.0 (4.1–6.0) 105	6.0 (5.1–7.1) 130
In Program^1^ %(CI) N Test				2.8* (2.1–3.6) 53	3.2* (2.4–4.0) 63	2.4 (1.8–3.2) 49	4.9 (4.0–5.9) 103	2.5 (1.9–3.3) 53	4.2 (3.5–5.2) 92
Outside Program %, (CI) N Test				4.2 (3.3–5.2) 79	2.2 (1.6–2.9) 44	3.9 (3.1–4.8) 78	2.3 (1.7–3.1) 49	2.5 (1.9–3.2) 52	1.7 (1.2–2.4) 38
NB Overall % (CI) N Test	2.8 (2.6–3.0) 1′080	2.8 (2.6–2.9) 1′059	2.7 (2.6–2.9) 1′073	2.3 (2.2–2.4) 927	2.1 (2.0–2.2) 872	1.8 (1.6–1.9) 752	1.7 (1.5–1.8) 724	1.4 (1.3–1.6) 647	1.1 (1.1–1.2) 534
c) Colonoscopy only									
Uri Overall, % (CI) N Test	4.1 (3.3–5.1) 77	4.3 (3.5–5.3) 80	6.0 (5.0–7.2) 113	3.2 (2.4–4.1) 60	6.2 (5.2–7.4) 124	4.0 (3.2–4.9) 80	4.1 (3.3–5.1) 86	4.8 (3.9–5.7) 100	4.8 (4.0–5.8) 105
In Program^2^ % (CI) N Test						0.9 (0.6–1.5) 19	1.1 (0.7–1.6) 22	1.0 (0.6–1.5) 21	1.3 (0.9–1.9) 29
Outside Program % (CI) N Test						3.0 (2.3–3.9) 61	3.1 (2.4–3.9) 64	3.8 (3.0–4.7) 79	3.5 (2.8–4.4) 76
NB Overall %, (Ci); N Test	3.7 (3.5–3.9) 1′426	4.3 (4.1–4.5) 1′666	4.5 (4.3–4.7) 1′751	4.8 (4.6–5.0) 1′922	5.5 (5.2–5.7) 2′272	5.9 (5.7–6.1) 2′504	6.5 (6.3–6.8) 2′855	6.3 (6.1–6.6) 2′850	6.8 (6.6–7.0) 3′152

Exclusion criteria: died, moved or changed insurance in that year. + included neighboring cantons: Glarus (GL), Lucerne (LU), Nidwalden (NW), Obwalden (OW), Schwyz (SZ).

1 in program = people included in the program who did not have to pay deductibles or co-pay. After 2014 billing for tests within the OSP used unique codes. 2 Colonoscopy inside/outside the program can only be determined after 2014; before FOBT was billed under the same code, whatever the setting. *Estimates from data provided by Uri’s Cantonal Office of Finance and its centralized database. In 2013, when the program was launched, the Canton of Uri provided free FOBT tests, without co-pay, so the bill was not sent to health insurances. Since 261 tests were reimbursed in 2013 and 20.2 % of the population of Uri was insured by CSS, we estimate 53 FOBT were performed within the program in 2013 and covered by the canton directly. The canton did not take over colonoscopy cost. In 2014, 304 total FOBT tests were performed and we estimated another 63 tests were performed outside the program.

In a multivariate adjusted logistic and multinomial regression model, we found that insurees in Uri were always more likely to have been tested for CRC than insurees in NBs (OR 1.49, [95%CI:1.36–1.63]) and more likely to have been tested with FOBT/both (OR 2.00, [95%CI:1.73–2.21]) and colonoscopy (OR 1.18, [95%CI:1.04–1.33]) (Table 3).

**Table 3:** Odds ratio of being tested for colorectal cancer within each calendar year for 50–69 year-old insurees, 2010 to 2018, CSS database. a) any test for colorectal cancer [fecal occult blood test (FOBT) or colonoscopy], b) any FOBT or FOBT and colonoscopy or c) any colonoscopy and no FOBT in Canton Uri compared to other neighboring cantons before and after the launch of the organized screening program in Uri in 2013.

	Testing Overall*		FOBT/both**		Colonoscopy Only**	
	OR	95% CI	OR	95 % CI	OR	95% CI
Uri^1^ (Ref = NB)	1.49	1.36–1.63	2.00	1.73–2.21	1.18	1.04–1.33
Time Period X Canton Interaction^2^	0.91	0.81–1.02	2.08	1.78–2.44	0.60	0.51–0.70
Gender (women)	1.10	1.07–1.13	1.11	1.06–1.17	1.09	1.06–1.13
Age^3^ (Ref = 50–59)	1.21	1.18–1.24	1.34	1.27–1.40	1.16	1.13–1.20
Residence (Ref = Urban)						
Intermediate	0.89	0.86–0.92	0.77	0.73–0.83	0.94	0.90–0.98
Rural	0.75	0.73–0.77	0.64	0.60–0.68	0.80	0.77–0.83
PCG^4^ N>=1 (Ref = None)	1.64	1.59–1.68	1.61	1.53–1.69	1.65	1.60–1.70
Managed Care Model^5^ (Ref = None)	1.06	1.03–1.09	1.17	1.11–1.23	1.02	0.98–1.05
Year^6^ (Ref = 2010)						
2011	1.09	1.03–1.15	1.00	0.92–1.08	1.16	1.08–1.24
2012	1.12	1.06–1.18	0.98	0.90–1.07	1.22	1.14–1.31
2015	1.18	1.12–1.24	0.62	0.56–0.68	1.60	1.49–1.70
2016	1.28	1.21–1.35	0.60	0.55–0.66	1.78	1.67–1.90
2017	1.20	1.14–1.26	0.50	0.45–0.55	1.73	1.62–1.85
2018	1.23	1.17–1.30	0.43	0.36–0.47	1.86	1.74–1.98

*OR of being tested withFOBT or colonoscopy versus no test for each calendar year. Results from multivariate adjusted logistic regression model adjusted for gender, age, PCGs, managed care model, Year considered, Canton Uri vs the other neighboring cantons. Interaction term added testing the interaction between period before the organized screening program (years 2011–2012 vs 2015.2018) and canton Uri vs the other cantons. Years 2013 and 2014 excluded given the lack of information about which specific insuree had had FOBT that was directly reimbursed, by Canton Uri and for which no bill was sent to health insurance.

**OR of being tested with FOBT (or FOBT and colonoscopy) or colonoscopy-only versus no test for each calendar year. Results from multivariate adjusted multinomial model adjusted for gender, age, PCGs, managed care model, Year considered, Canton Uri vs the other neighboring cantons. Interaction term added testing the interaction between period before the organized screening program (years 2011–2012 vs 2015.2018) and canton Uri vs the other cantons. Years 2013 and 2014 excluded given the lack of information about which specific insuree had had FOBT that was directly reimbursed, by the Canton Uri and for which no bill was sent to health insurance. 1: canton of living = Uri. Comparison cantons: Glarus (GL) Lucerne (LU), Nidwalden (NW), Obwalden (OW), Schwyz (SZ); 2: Interaction between time (2010–2012 versus 2015–2018) and location (Uri) 3: Age 60–69 versus 50–59; 4: pharmacy-based-cost-groups 5: following models: HMO, Telemedicine, family physician; 6: 2013 and 2014 were excluded due to missing billing in claims data.

We compared testing in Uri before and after the OSP was initiated (2010–2012 vs 2015–2018) to testing in NBs (interaction term between time periods and canton) and found Uri insurees were more likely to have been tested with FOBT/both (OR 2.08 [95%CI:1.78–2.44]) and less likely to have had colonoscopies (OR 0.60 [95%CI:0.51–0.70]) after the OSP start. Overall testing rates did not change (Table 3). The results did not differ significantly in stratified analyses by gender. Other covariates significantly associated with testing for both methods were being a man, being 60–69 years old, living in an urban region, and having more than 1 PCG.

### 3.2 Proportion up-to-date with CRC testing in 2018

We included 12′838 continuously insured insurees in our analysis of the proportion up-to-date with CRC testing: 600 for Uri and 12′238 for NB (Appendix Table 6).

In 2018, 42.5% of eligible Uri insurees were up-to-date with CRC testing; 9.2% were billed a FOBT/both in 2018 or 2017 and 35.7% were billed for a colonoscopy only within the last 9 years. In NBs, 40.7% were up-to-date with CRC testing; 2.7% had had an FOBT/both, and 39.0% had had a colonoscopy (Appendix Table 7).

In multivariate adjusted analyses, insurees in Uri were more likely to be up-to-date with FOBT/both (OR 3.78 [95%CI:2.84–5.02]) than insurees in NB, but less likely to be up-to-date with colonoscopy (OR 0.81 [95%CI:0.68–0.95]). There was no significant difference in being up-to-date overall. Other covariates significantly associated with being up-to-date with testing were being a man, being 65–69 years old, living in urban areas, and having more than one PCG (Appendix Table 8).

## 4 Discussion

### 4.1 Summary

Our analysis of claims data from a large health insurance revealed that yearly CRC testing rates between 2010 and 2018 were always higher in Uri than in NBs, even before the OSP implementation. Testing rates increased over time in all regions. The trend in FOBT testing was upward in Uri and downward in NBs. The OSP implementation was significantly associated with higher FOBT testing rates in Uri. The downward trend was evident in FOBT tests outside the Uri program same as in NBs, which indicates that improved screening rates within the OSP likely account for the overall increase in FOBT (Fig. 1). Over time, colonoscopy rates increased in Uri and NBs, but they increased less in Uri. Overall testing was not significantly higher in Uri, but more people in Uri were up-to-date with FOBT in 2018.

### 4.2 Direct implications

In the context of Uri, were colonoscopy and overall testing rates were already relatively high and a new OSP offered participants a choice of CRC screening tests, we found that the FOBT rate increased more steeply than the colonoscopy rate. The OSP in Uri automatically sends information that helps eligible participants make an informed choice between FOBT and colonoscopy, which may explain the increase in FOBT rates (Gupta, 2013, Adler et al., 2014, Inadomi, 2012). The OSP also waived deductible and co-pay for CRC tests performed within the program, which might also have contributed to increases in testing. (Wharam et al., 2016) The slight downward trend in colonoscopy rates after implementing an OSP runs counter the Swiss trend, perhaps because a study conducted in 2000 raised public awareness and, possibly, already enhanced colonoscopy rates (Marbet et al., 2008). Since colonoscopies previously performed outside the Uri program are now performed within it, this may indicate a stable rather than decreased colonoscopy rate (Fig. 2).

Studies comparing regions before and after an OSP start or across regions are scarce. A study in France compared the likelihood of having had an FOBT between one area with and one without an OSP and found an OR of 3.9 (Eisinger et al., 2008). A New York City study evaluated a CRC screening initiative. The percentage of eligible residents up-to-date with screening before implementation was 42% and afterwards climbed to 70% (Itzkowitz et al., 2016). In international comparisons of OSP screening rates, OSP structure and participation rates vary widely (Klabunde et al., 2015). Germany and Austria have OSPs and about 60–70% of their eligible population is up-to-date with screening (Cardoso et al., 2020, Chen et al., 2019). Coverage in Uri was 42.5% among CSS insurees by 2018—lingering below the EU guideline goal for OSPs of at least 45% screening uptake in the eligible population.

We know from previous studies, that OSPs have the potential to enhance testing rates and therefore lower CRC mortality. OSPs have devised various strategies to increase program participation. Giving people an informed choice of testing strategies is one option. Testing rates may also rise if an OSP reminds eligible participants or their health care providers to schedule and attend screening appointments and then follows up if individuals do not respond. OSPs can also remind participants to repeat a test or send FOBT-kits directly to their home (DeGroff et al., 2018, Selby, 2022). The Netherlands, the USA, and other countries have tested such interventions and reached CRC testing rates to about 65–70% in the eligible population (DeGroff et al., 2018; Dutch National Institute for Public Health: Overview of quality assurance within the CRC screening program, 2020). However, we believe the proportion of the population having already been tested for CRC through opportunistic screening is important to consider when analyzing the participation rate to an OSP. In Uri, significant proportions of the population had already been tested therefore reducing the eligible population to the OSP. The Uri program sends an invitation letter to each resident when they turn 50. The letter describes the program and asks them to choose between a FOBT or a colonoscopy. People who complete a FOBT are mailed their next test kit two years later. But Uri does not remind non-responders or send systematic reminders to primary care physicians.

### 4.3 Limitations

Our study has several potential limitations that we made efforts to mitigate. Three gaps in claims data might have caused our estimates to deviate from true yearly testing rates. Regarding FOBT numbers, we have no insurance bills for FOBTs performed within the OSP in 2013 and 2014 and instead substituted data from the OSP and estimated testing rates. We knew that the OSP data might less accurately report the number of FOBTs than claims data, so we excluded 2013 and 2014 from our logistic regression model. In addition, we cannot distinguish between different kinds of FOBT testing in our dataset. Evidence from the UK suggest that changing from gFOBT to iFOBT was associated with increased participation (Clark et al., 2021). We were not able test this hypothesis based on the claims data available.

Second, our claims data omitted inpatient tests. We had earlier estimated in another claims dataset that between 3.5 and 5% of colonoscopies in Switzerland are performed on inpatients (Schneider et al., 2021). When conducting additional estimations with this data, we found that in Uri, inpatient colonoscopies accounted for 7.4% of colonoscopies. We thus may have underestimated colonoscopy rates and overall testing rates in Uri.

Third, when the Uri OSP began, it excluded those who participated in the 2000–2001 cohort study because they had already been offered free colonoscopies and FOBTs. This exclusion criterion was later rescinded, but we estimate that, in 2013, between 100 and 150 eligible people were insured by CSS and excluded from the Uri OSP. This exclusion may have artificially lowered the colonoscopy rate in the early years of the Uri program. Participants who underwent colonoscopy in 2000/2001 might also be less willing to undergo a second colonoscopy. We assume some of them would be in our dataset and this factor could also lower the colonoscopy rate in Uri during our study period.

Finally, claims data often underestimates testing rates because it misses tests for which participants did not enter claims. We thus balanced it with SHS data to compare our retrieved rates. Survey data tends to overestimate testing by recall and reporting bias. As expected, we found a slightly higher proportion of the eligible population up-to-date with testing in the SHS data than in the CSS dataset. The slight difference had little effect on our study because, overall, testing rates were similar and FOBT rates were still higher in Uri than NB (Appendix Table 7). The much-narrowed confidence intervals in the more powered CSS dataset is a clear strength of our method and enabled to detect significant differences between cantons with small population sizes.

## 5 Conclusion

Because evaluating the effects of OSPs on testing rates is a precursor to effectively raising them and maintaining them, we encourage insurance companies to provide national datasets and suggest independent researchers use these datasets to track the effect of OSPs on testing rates. Since a decline in mortality can only be detected many years after implementing a CRC screening program, we need to collect informative data earlier. As a proxy for mortality, we can measure polyp numbers and monitor cancer stages in the larger cantons (Uri is too small for that) and use national numbers to compare the effect of OSPs across countries. In all cantons, we can test interventions, e.g., send FOBT kits directly to people’s homes or use reminder systems, to determine if they increase testing rates. Our finding that FOBT rates increased in Uri after it implemented an OSP supports the claim that offering people an informed choice will raise the FOBT rate and may increase overall screening rates.

## Funding

Our work was funded by the Swiss Cancer Research Foundation, Health Services Research (HSR-4366-11-2017) and the Swiss National Science Foundation, National Research Program 74 (NFP74 407440-167519) “Smarter Health Care”. Funders played no role in designing or conducting the study. They did not participate in collecting, managing, analyzing, or interpreting the data, or in preparing, reviewing, or approving the manuscript.

## Declaration of Competing Interest

CS is employed by the Research Center of the Swiss health insurance company CSS. The remaining authors declare that they have no known competing financial interests or personal relationships that could have appeared to influence the work reported in this paper.
