# Supplementary material for: Change in colorectal cancer (CRC) testing rates associated with the introduction of the first organized screening program in canton Uri, Switzerland: Evidence from insurance claims data analyses from 2010 to 2018
Source: Prev Med Rep. 2022 Jun 10;28:101851. doi: 10.1016/j.pmedr.2022.101851 (PMC9218582; doi:10.1016/j.pmedr.2022.101851)
Supplement: Supplementary data 1 [file mmc1.docx]

**Appendix**

**Change in colorectal cancer (CRC) testing rates associated with the introduction of the first organized screening program in Canton Uri, Switzerland: Evidence from insurance claims data analyses from 2010-2018**

Sarah Bissig^1^, Lamprini Syrogiannouli^1^, Rémi Schneider^1^, Kali Tal^1^, Kevin Selby^2^, Cinzia Del Giovane^1^, Jean-Luc Bulliard^2^, Oliver Senn^3^, Cyril Ducros^2^, Christian P.R. Schmid^4^, Urs Marbet^5^, Reto Auer^1,2^

1 Institute of Primary Health Care (BIHAM), University of Bern, Bern, Switzerland

2 Center for Primary Care and Public Health (Unisanté), University of Lausanne, Lausanne, Switzerland

3 Institute of Primary Care, University and University Hospital of Zurich, Zurich, Switzerland

4 CSS Institute for Empirical Health Economics, Tribschenstrasse 21, Lucerne, Switzerland

5 Division of Gastroenterology and Hepatology Cantonal Hospital of Uri, Altdorf, Switzerland

**Corresponding Author**

Reto Auer, MD, MAS

Assistant Professor of Primary Care

Head of Research

Institute of primary health care (BIHAM), University of Bern

Mittelstrasse 43

CH - 3012 Bern

[reto.auer@biham.unibe.ch](mailto:reto.auer@biham.unibe.ch)

+41 31 631 58 79

# Appendix

**Appendix Table 1**: Abbreviations used in the Manuscript

| AL | Swiss analysis list for laboratory measures |
| --- | --- |
| CRC | Colorectal cancer |
| FIT | fecal immunochemical test, also called iFOBT |
| FOBT | fecal occult blood test; guaiac or immunochemical |
| FSO | The Federal Statistics Office |
| gFOBT | guaiac-based FOBT |
| iFOBT | immunochemical FOBT, also called FIT |
| NB | neighboring cantons |
| OSP | Organized colorectal cancer screening program |
| PCG | pharmacy-based cost group |
| SHS | Swiss Health Interview Survey |
| TARMED | Swiss ambulatory procedures codes |
| Uri | the Canton of Uri |

| **Source** | **Content** | **Available Years** |
| --- | --- | --- |
| CSS health insurance basic costumer data | Sociodemographic factors, health care plan, billing costs | 2010-2018 |
| TARMED* billing codes in CSS health insurance claims data | Billed colonoscopies and sigmoidoscopies | 2010 -2018 |
| AL** billing codes in CSS health insurance claims data | Billed FOBTs | 2010-2018 (in 2013 and 2014, bills taken over by program directly) |
| Surveillance data from the organized screening program in Uri | Amount of additional free FOBT not billed to health insurances | 2013, 2014 |
| Swiss Health Interview Survey dataset | Comparison dataset for percentage up to date with CRC screening | 2017 |

**Appendix Table 2: Overview of used data sources**

**Appendix Table 2**: Overview over the used data sources. *Tarmed: billing codes for ambulant procedures used in Switzerland. **AL: billing codes used laboratory testing used in Switzerland

**Appendix Table 3**: Extracted CRC testing billing codes from insurance claims data

| **Catalog** | **Code** | **Description** |
| --- | --- | --- |
| TARMED* | 19.06 | colonoscopy |
| TARMED | 19.07 | Sigmoidoscopy |
| Tarifcode** | 0001.1220.002 | colonoscopy with biopsy program UR |
| Tarifcode | 0001.1221.002 | colonoscopy-only program UR |
| Tarifcode | 0001.1222.002 | colonoscopy with polypectomy program UR |
| AL*** | 1583.00 | occult blood |
| AL | 1583.01 | occult blood |
| Tarifcode | 0001.1234.001 | FOBT in program UR |

**Appendix Table 3:** . *Tarmed: billing codes for ambulant procedures used in Switzerland. **Tarifcode: agreed billing codes between the OSP organizers and the health insurances for screening within the program in use after 2014. ***AL: billing codes used laboratory testing used in Switzerland

**Appendix Table 4** Overall excluded population

| **Year** | **Excluded population** |
| --- | --- |
| 2010 | 10411 |
| 2011 | 9838 |
| 2012 | 9503 |
| 2013 | 9082 |
| 2014 | 8801 |
| 2015 | 8576 |
| 2016 | 7969 |
| 2017 | 8203 |
| 2018 | 8571 |

**Appendix Table 4:** Overall excluded population in the included cantons (GL, LU, NW, OW, SZ, UR) for each year from 2010-2018. Reasons of Exclusion: a)change in insurance b) death c)moving

**Appendix Table 5**: Calculation details for additional free FOBT testing, corrected colonoscopy rates and overall testing rates in Uri 2013/2014

|  | **Parameter** | **Data origin / calculation** | **Year=2013** |
| --- | --- | --- | --- |
|  | **Uri overall** |  |  |
| 1 | Population 50-69 in Uri (N) | Bfs database | 9390 |
| 2 | Free FOBT performed in Uri (N) | Uri program data | 261 |
| 3 | Free FOBT with following colonoscopy* (N; %) | Uri program data | 14; 5.2% |
|  | **CSS insurees in Uri** |  |  |
| 4 | Population 50-69 in dataset (N) | CSS claims data | 1897 |
| 5 | CSS market share in Uri (%) | A4/A1 | 20.2% |
| 6 | Estimated free FOBT CSS insurees (N) | A2*A5 | 53 |
| 7 | Estimated free FOBT rate CSS insurees (%) | A6/A4 | 2.8% |
| 8 | Estimated free FOBT + Colo CSS insurees* (N) | A5*A3 | 3 |
| 9 | Corrected absolute colo testing* (N) | N colos in CSS claims data -A8 | 60 |
| 10 | Corrected relative colo testing rate (%) | A9/A4 | 3.2% |
| 11 | Estimated overall testing rate CSS insurees (%) | claims data rates + A7 + A10 | 10.1% |

**Appendix Table 5** Calculation details for estimating free FITs done in 2013 and 2014 in Uri. Data extracted from CSS claims data and data from the Uri program. Calculation showing 2013 data, same procedure for 2014 data.

*Percentage of people that were billed for a colonoscopy in claims data after a free FOBT in the program, according to program data. These people should be counted in the FOBT group. Data is based on reported colonoscopies performed after FOBT, according to program data.

**
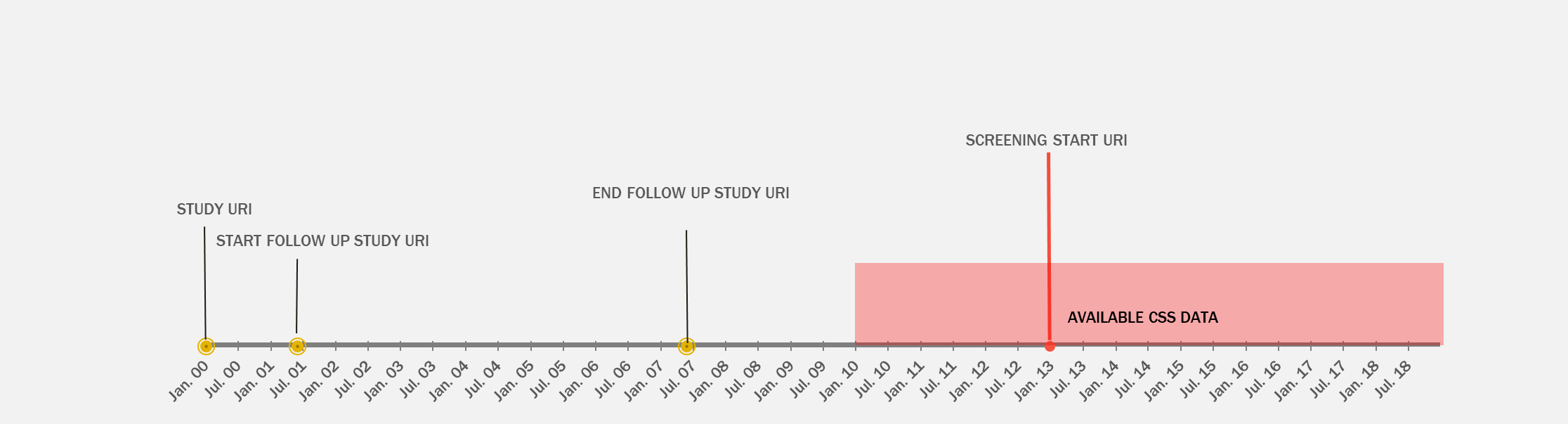
**

**Appendix Figure 1:** Timeline of events in Uri. Study in Uri was a closed cohort study examining the effectiveness of colonoscopy and FOBT in preventing CRC and its pre-stages and accessing the participants’ preferences for CRC screening method.

**Appendix Figure 2**: Overall and method-specific yearly testing rates in UR and NBs from 2010-2018; CSS database

***Appendix Figure 2:*** *Exclusion criteria a) moved b) died c) changed insurance each respective year. + included neighboring cantons: Glarus (GL), Lucerne (LU), Nidwalden (NW), Obwalden (OW), Schwyz (SZ). Data for 2013 and 2014: Estimates from data from the Cantonal Office of Finance from Uri and from the centralized database from Uri. In 2013, when the program was launched, the FOBT tests were taken over for free and without co-pay from the Canton of Uri. No bill was sent to health insurances. Given the 261 tests reimbursed in 2013 and 20,2 % of the population of Uri insured by CSS, 53 tests were estimated to have been performed in 2013 in study population that were taken over by the canton directly. No colonoscopy costs taken over by canton directly. For 2014, 304 total FOBT tests performed, 63 estimated in the population.*

**Appendix Table 6:** Descriptive statistics of included population between 59-69 in Uri and neighboring cantons^+^ (NB) in the SHS* dataset in 2017 and in the CSS dataset in 2018 for estimating proportion up-to-date with testing; SHS database 2017 and CSS data base 2018

|  | CSS 2018 | | SHS 2017 | |
| --- | --- | --- | --- | --- |
|  | Uri | Uri | NB^+^ | NB |
| Population (N) | 600 | 94 | 399 | 12’238 |
| Gender (=woman) % (95%CI) | 52.3(48.3-56.3) | 57.5 (46.6-67.6) | 50.2 (43.9-56.5) | 51.0(50.1-51.9) |
| Age % (95%CI) |  |  |  |  |
| 59-64 | 64.3(60.4-68.1) | 60.1 (49.3-70.0) | 62.8 (56.7-68.5) | 66.2(65.3-67.0) |
| 65-69 | 35.7(31.9-39.6) | 39.9 (30.0-50.7) | 37.2 (31.5-43.3) | 33.8(33.0-34.7) |
| Residence^1^ % (95%CI) |  | -- | -- |  |
| Urban | 66.7(62.8-70.3) | -- | -- | 48.4(47.5-49.3) |
| Intermediate | 11.5(9.2-14.3) | -- | -- | 21.2(20.5-21.9) |
| Rural | 21.8(18.7-25.3) | -- | -- | 30.4(29.6-31.2) |
| Managed care ^2^ (=Yes) % (95%CI) | 52.0(48.0-56.0) | 53.2 (42.5-63.6) | 55.8 (49.5-62.0) | 61.5(60.6-62.3) |
| PCG >= 1 ^3^ % (95%CI) | 42.0(38.1-46.0) | -- | -- | 46.4(45.5-47.3) |

**Appendix Table 6** CSS database: people were excluded if: a) they moved, b) died c) changed their insurance sometime between 2010 and 2018. + = included neighboring cantons: Glarus (GL), Lucerne (LU), Nidwalden (NW), Obwalden (OW), Schwyz (SZ)

* a 5-yearly survey on health issues conducted in a randomly chosen sample representing the Swiss population -- not available in the SHS dataset

1 determined using the zipcode and a list provided by the Federal statistical office of Switzerland (FSO) 2 including following models: family physician, HMO, telemedicine 3 pharmacy-based cost group; 3 tool to assess chronic health conditions using information on medication

## Comparison with the SHS 2017 data set

SHS’s sampling scheme includes a representative number of participants per canton. In our population of interest, SHS 2017 included 94 participants from Uri and the CSS dataset included 600; SHS 2017 included 399 participants from NB and the CSS dataset included 12’238 (Appendix Table 5). In Uri, 54.1% (95%CI 43.5-64.3) of participants reported they were up-to-date with CRC testing; 13.5% (95%CI 7.7-22.5) with FOBT/both and 47.0% (95%CI 36.1-58.2) with colonoscopy. In NBs, 52.5% (95%CI 46.2-58.7) reported they were up-to-date with testing; 9.1% (95%CI 5.9-13.8) with FOBT and 46.7% (95%CI 40.3-53.1) with colonoscopy.

**Appendix Table 7:** Percentages of population up-to-date with testing in SHS^#^ population in 2017 and CSS population in 2018 respectively, aged 59 to 69 for Uri and neighboring cantons; SHS database 2017 and CSS database 2018

|  | | Testing overall* | | FOBT or both** | | Colonoscopy only*** | | |
| --- | --- | --- | --- | --- | --- | --- | --- | --- |
|  | | **Uri** | **NB** | **Uri** | **NB** | **Uri** | | **NB** |
| % in SHS 2017.  (CI);  N tested/ N total | 54.1  (43.5-64.3)  49/82**** | | 52.5  (46.2-58.7)  214/369**** | 13.5  (7.7-22.5)  12/82**** | 9.1  (5.9-13.8)  37/369**** | 47.0  (36.1-58.2)  37/82**** | 46.7  (40.3-53.1)  177/369**** | |
| % in CSS 2018. (CI);  N tested/ N total | 42.5  (38.5- 46.6)  255/600 | | 40.7  (39.8- 41.5)  4’975/12’238 | 9.2  (7.0-11.8)  55/600 | 2.7  (2.5-3.0)  335/12’238 | 35.7  (31.8-39.6)  214/600 | 39.0  (38.2-39.9)  4’775/12’238 | |

**Appendix Table 7** # a 5-yearly survey on health issues conducted in a randomly chosen sample representing the Swiss population. Weighted percentages. Participants in Uri with complete data. 82; Participants in NB with complete data: 369.

*any CRC test in recommended interval (FOBT in last 2 years or colonoscopy in last 9 years [CSS] or last 10 years [SHS])

**FOBT only in last 2 years or FOBT in last 2 years and colonoscopy in last 9 years (CSS)/ 10 years (SHS)

*** colonoscopy in last 9 years (CSS) / 10 years (SHS) and no FOBT in last 2 years

**** Number of participants in the SHS. SHS weights participants to obtain weighted percentages. Weighted percentages therefore don’t necessarily match the computed percentage based on source data presented.

**Appendix Table 8:** Odds ratio of being up-to-date with testing in 2018 (colonoscopy within last 9 years or FOBT within last 2 years for 58-69 year-old insurees comparing population living in Canton Uri to those living in other neighboring cantons; CSS dataset 2018. a) any colonoscopy within last 9 years or FOBT within last 2 years, b) any FOBT within last 2 years with or without colonoscopy within last 9 years or c) any colonoscopy within last 9 years and no FOBT within last 2 years

|  | a) Testing overall* | | b) Fobt or both** | | c) Colonoscopy only** | |  |
| --- | --- | --- | --- | --- | --- | --- | --- |
|  | OR | 95% CI | OR | 95 % CI | OR | 95% CI | |
| Canton Uri (ref=NBs^+^) | 1.03 | 0.88-1.21 | 3.78 | 2.84-5.02 | 0.81 | 0.68-0.95 | |
| Gender (ref= woman) | 1.09 | 1.02-1.16 | 1.28 | 1.05-1.54 | 1.07 | 1.00-1.14 | |
| Age^1^ (ref=58-64) | 1.12 | 1.04-1.21 | 1.16 | 0.94-1.42 | 1.11 | 1.03-1.20 | |
| Residence^2^ (ref=urban) |  |  |  |  |  |  | |
| Intermediate | 0.87 | 0.80-0.95 | 0.85 | 0.66-1.11 | 0.87 | 0.80-0.96 | |
| Rural | 0.70 | 0.65-0.76 | 0.74 | 0.59-0.94 | 0.71 | 0.66-0.77 | |
| PCG n>=1^3^ (ref=none) | 1.58 | 1.48-1.69 | 1.44 | 1.19-1.76 | 1.56 | 1.46-1.67 | |
| Managed care model^4^ (ref=none) | 1.07 | 1.00-1.15 | 1.14 | 0.93-1.39 | 1.05 | 0.98-1.12 | |

**Appendix Table 8:** Exclusion criteria: a)moved b) died c) changed insurance any time during the 9-year period. * OR of being tested for FOBT within the last 2 years or colonoscopy-only within last 9 years for inhabitants of Uri living in this canton for the whole 9 years considered compared to those living continuously in neighboring cantons. Results from multivariate adjusted logistic regression model adjusted for gender , age, PCGs,, managed care model, Canton Uri vs the other neighboring cantons. ** OR of being tested for FOBT (or FOBT and colonoscopy) within the last 2 years or colonoscopy-only within last 9 years for inhabitants of Uri living in this canton for the whole 9 years compared to those living continuously in neighboring cantons. Results from multivariate adjusted multinomial model adjusted for gender , age, PCGs, managed care model, Canton Uri vs the other neighboring cantons.

1 Reference= 59-64 compared to 65- 69 year-olds; 2 determined using the zipcode and a list provided by the Federal statistical office of Switzerland (FSO) 3 pharmacy-based cost group; tool to assess chronic health conditions using information on medication for the four following models: HMO, Telemedicine, family physician + canton of living = Uri; included neighboring cantons: Glarus (GL), Lucerne (LU), Nidwalden (NW), Obwalden (OW), Schwyz (SZ).
